# Flexible integration of visual cues in adolescents with autism spectrum disorder

**DOI:** 10.1002/aur.1509

**Published:** 2015-06-19

**Authors:** Rachael Bedford, Elizabeth Pellicano, Denis Mareschal, Marko Nardini

**Affiliations:** ^1^ Biostatistics DepartmentInstitute of Psychiatry, King's College LondonUnited Kingdom; ^2^ Centre for Research in Autism and Education (CRAE)Institute of Education, University of LondonUnited Kingdom; ^3^ School of PsychologyUniversity of Western AustraliaPerthAustralia; ^4^ Centre for Brain and Cognitive DevelopmentBirkbeck University of LondonUnited Kingdom; ^5^ Department of PsychologyDurham UniversityDurhamUnited Kingdom

**Keywords:** autism, adolescent, vision, depth perception, cue integration, fusion

## Abstract

Although children with autism spectrum disorder (ASD) show atypical sensory processing, evidence for impaired integration of multisensory information has been mixed. In this study, we took a Bayesian model‐based approach to assess within‐modality integration of congruent and incongruent texture and disparity cues to judge slant in typical and autistic adolescents. Human adults optimally combine multiple sources of sensory information to reduce perceptual variance but in typical development this ability to integrate cues does not develop until late childhood. While adults cannot help but integrate cues, even when they are incongruent, young children's ability to keep cues separate gives them an advantage in discriminating incongruent stimuli. Given that mature cue integration emerges in later childhood, we hypothesized that typical adolescents would show adult‐like integration, combining both congruent and incongruent cues. For the ASD group there were three possible predictions (1) “no fusion”: no integration of congruent or incongruent cues, like 6‐year‐old typical children; (2) “mandatory fusion”: integration of congruent and incongruent cues, like typical adults; (3) “selective fusion”: cues are combined when congruent but not incongruent, consistent with predictions of Enhanced Perceptual Functioning (EPF) theory. As hypothesized, typical adolescents showed significant integration of both congruent and incongruent cues. The ASD group showed results consistent with “selective fusion,” integrating congruent but not incongruent cues. This allowed adolescents with ASD to make perceptual judgments which typical adolescents could not. In line with EPF, results suggest that perception in ASD may be more flexible and less governed by mandatory top‐down feedback. ***Autism Res*** *2016, 9: 272–281*. © 2015 International Society for Autism Research, Wiley Periodicals, Inc.

## Introduction

Unusual sensory responses in autism spectrum disorder (ASD) were first described by Kanner [1943], involving both hypo‐ and hyper‐responsiveness to sensory stimuli. More recently, the importance of sensory sensitivities has been emphasized by their addition to autism diagnostic criteria [Diagnostic and Statistical Manual 5th edition; DSM‐5, American Psychiatric Association, 2013; see Pellicano, 2013 for a recent review]. Iarocci and McDonald [2006] have argued that multisensory perception and sensory integration may offer a useful way to conceptualize sensory processing in autism. This study examines information integration during visual processing in adolescents with ASD.

In children with autism, early evidence from the McGurk task (in which a visually presented /ga/ is paired with an auditory /ba/ causing the intermediate phoneme /da/ to be perceived) suggested that cross‐modal integration was reduced [DeGelder, Vroomen, & Van der Heide, 1991]. Subsequently, similar audio‐visual (AV) integration difficulties have been found across a range of studies using complex social stimuli [e.g., Bebko, Weiss, Demark, & Gomez, 2006; Megnin et al., 2012; Smith & Bennetto, 2007]. However, Mongillo et al. [2008] found AV integration impairments in autism for human face/voice perception, but not for nonsocial stimuli (e.g., bouncing ball). Indeed, a growing body of recent studies using low‐level cues such as beeps and flashes, find no evidence for a sensory integration deficit in individuals with ASD [Foss‐Feig et al., 2010; Keane, Rosenthal, Chun, & Shams, 2010; Kwakye, Foss‐Feig, Cascio, Stone, & Wallace, 2010; Magnée, Oranje, van Engeland, Kahn, & Kemner, 2009; Mongillo et al., 2008; Van der Smagt, van Engeland, & Kemner, 2007].

The majority of these previous studies focus on temporal integration of AV cues, motivated by the observation of sensory atypicalities in autism, rather than testing model‐based predictions. By taking a Bayesian approach, well‐tested in typical development, we are able to apply a rigorous methodology to address the question of cue integration in autism. In typical development, abilities to compare and combine sensory signals develop on a range of time‐scales. For example, there is evidence for cross‐modal interactions in newborns [Streri, 2012], for postnatal experience‐dependent development of AV integration for spatial orienting [Neil, Chee‐Ruiter, Scheier, Lewkowicz, & Shimojo, 2006; Wallace & Stein, 1997] and for notably late development of abilities to improve perceptual precision by integrating multiple cues [Nardini, Jones, Bedford, & Braddick, 2008; Gori, Del Viva, Sandini, & Burr, 2008]. Children show no improvement in the precision of spatial estimates by combining visual and non‐visual cues to location [Nardini et al., 2008] or visual and haptic cues to form until after 8 years [Gori et al., 2008].

**Figure 1:**
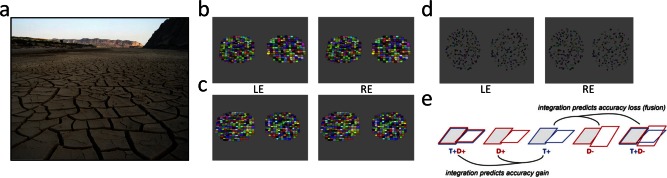
(a) Texture provides information about 3D layout—here about the angle (slant) of the ground relative to the viewer (camera). A stereoscopic view would also provide disparity information, a second independent cue to the surface slant. (**b)** Example left‐eye (LE) and right‐eye (RE) views of stimulus pair in condition *T+D+*. Both texture and disparity indicate that the left‐hand plane has the greater slant toward the horizontal. The stimuli may be seen in stereo by free fusion, but disparities are only correct when the display takes up 13° degrees of visual angle, as in the experiment. Monocular viewing of only one eye's view (e.g., LE) corresponds to the texture‐only (*T+*) condition. (**c)** Example stimulus pair in condition *T+D‐*. While texture indicates that the left‐hand plane has the greater slant toward the horizontal, disparity indicates that it has the less. (**d)** Example stimulus pair in condition *D+*. There is no useful texture information, but disparity indicates that the left‐hand plane has the greater slant toward the horizontal. (**e)** Schematic view of conditions and the relationships predicted by integration of cues. In each condition, participants judge whether the 45° slanted standard (grey, shown left) has same or different slant as a comparison slanted 45 ± 12.5°, based on different cues (only the “different” case—as seen on half of trials—is illustrated). Photo in (**a)** from https://www.flickr.com/photos/10709229@N00/2101324396/under the creative commons license.

Even within the single modality of vision, mature cue integration for judging the slant of a surface using two depth cues does not develop until 12 years of age [Nardini, Bedford, & Mareschal, 2010]. Seeing in depth relies on multiple cues including stereoscopic disparity, motion, texture, and shading [Howard & Rogers, 2008]. For example, a regular texture on a surface (Fig. 1a) provides useful information about its 3D layout. Nardini et al. [2010] investigated children's integration of binocular disparity and texture gradient information to judge whether two surfaces had the same or different slant. When the slants were different, this was evident via either single or combined cues, and combined cues were sometimes in agreement with each other (congruent) and sometimes in disagreement (incongruent). With congruent combined cues, adults’ ability to judge slant was improved by having the two cues together over either one alone. This benefit of combining (averaging) sensory estimates can be explained by a reduction in sensory noise or uncertainty [Ernst & Banks, 2002; Hillis, Watt, Landy, & Banks, 2004]. The underlying principle is one familiar from statistical testing: because data contains random noise, estimates are more reliable when multiple data points are averaged. Similarly, perceptual estimates can be improved by averaging. Yet when the two visual cues conflict and signal different slants, estimating slant by taking an average across cues can make slant differences between the two stimuli appear less than they are when judged via single cues. In line with this, Nardini et al. [2010] found that with incongruent combined cues, adults’ precision was reduced. Adults could not help but average the cues, even when this made them worse at the task than just relying on single cues—an effect termed “mandatory fusion” [Hillis, Ernst, Banks, & Landy, 2002; Prsa, Gale, & Blanke, 2012]. Typically developing 6‐year‐olds showed a different pattern: no mandatory fusion. They did not gain an accuracy benefit by integrating congruent cues, but their ability to keep cues separate also allowed them to remain good at slant judgments for stimuli in which the cues were incongruent [Nardini et al., 2010].

An observer's expectation that certain cues go together and so should be averaged has been described and modeled in terms of a “coupling prior”—a probability distribution describing how likely it is that two cues will signal the same value [Ernst, 2006; Ernst, 2012; Ernst & Di Luca, 2011]. A relatively flat coupling prior^1^ leads to the ability to keep cues separate, whereas a relatively peaked one leads to their mandatory combination (or fusion). The extent of mandatory fusion varies, for example, it is less between modalities (vision and touch) than within a modality (two visual cues) [Hillis, et al., 2002]. This suggests that observers use different coupling priors, specific to different combinations of cues. The fact that young children do not show mandatory fusion of two visual cues suggests that in typical development, as the visual processing system matures, it is still acquiring “coupling priors” for which cues go together.

In this study, we used the same approach as Nardini et al. [2010] to measure the abilities of typical and autistic adolescents at comparing the 3D slants of surfaces using disparity and texture information (example stimuli, Fig. 1b–d). In autism, aspects of visual processing of texture and binocular disparity have previously been studied in isolation. Detecting the orientation of a pattern defined by texture (second‐order grating) is impaired in children with autism [Bertone, Mottron, Jelenic, & Faubert, 2003, 2005], despite typical or even enhanced performance for luminance‐defined (first‐order) gratings. Similarly, behavioral data from Vandenbrouke, Scholte, van Engeland, Lamme, and Kemner [2008] suggest that boundary detection may be impaired in autism. In the domain of binocular vision, the rate of perceptual alternation during binocular rivalry has been shown to be slower in autism [Robertson, Kravitz, Freyberg, Baron‐Cohen, & Baker, 2013], although Said, Egan, Minshew, Behrmann, and Heeger [2013] found no difference between autistic and control participants. These studies investigated relatively low‐level aspects of texture and binocularity, while this study investigates the specific combination of texture and binocular information to make perceptual judgments about 3D layout. It is possible that these judgments of 3D shape may also be atypical in autism, but previous findings do not clearly predict that participants with autism should be impaired when making slant judgments based on either or both cues. To check that ASD and typical groups can indeed use both cues singly to make 3D judgments (as well as to test how they use them in combination), this study includes single‐cue as well as combined‐cue conditions.

The rationale for using this task in autism was to test integration abilities within a rigorous Bayesian framework that has been developed in the typical literature. A strength of the approach is that by testing performance with single vs. combined cues, it is able to address when and how visual information is integrated, and the extent to which integration is atypical in the ASD group. A strength of these particular stimuli is that they show documented development and maturation in typical children [Nardini et al., 2010], which can be compared with the present pattern of results. The fact that in typical development, even within‐modality cue integration follows a protracted trajectory raises the possibility that, over the course of development, cue integration could be at particular risk of disruption in individuals with ASD. By simultaneously assessing performance when the cues conflict this study will enable us to tease apart different theoretical accounts of sensory processing in autism.

Sensory deficits in ASD have been proposed to reflect weaker “perceptual priors”—that is, a weaker influence of prior expectations on current percepts [Pellicano & Burr, 2012]. In the present framework, this might also predict a flatter “coupling prior,” representing a broader range of possibilities for how cues might go together than the narrower coupling prior leading to mandatory fusion of cues in adult controls. If so, we might see that individuals with ASD do not integrate either congruent or incongruent cues, similar to performance in 6‐year‐old typical children.

An alternative framework argues for enhanced perceptual functioning (EPF) autism [Mottron & Burack, 2001; Mottron, Dawson, Soulières, Huber & Burack, 2006], stating that while autistic individuals show increased attention to detail, performance in global and configural processing tasks is typical [Mottron, Burack, Stauder, & Robaey 1999]. The enhanced perceptual processing account argues that bottom‐up processes are superior in autism, leading to enhanced lower‐level processing. In addition, they propose a reduced influence of what they refer to as top‐down processing (i.e., decreased feedback from higher order visual cortical areas back to primary visual cortex) which leads to more “flexible” perception than is seen in typical development. Thus, EPF would predict typical integration when cues are congruent, but a flexible ability to keep them separate when they are incongruent.

In this study, we assess *within‐modality* integration of congruent and incongruent texture and disparity cues to judge slant in adolescents with ASD. We chose to look at 12‐ to 16‐year‐olds as we know from the typical literature that cue integration abilities mature to adult‐like performance by 12 years [e.g., Nardini et al., 2010]. There are three possible patterns of performance that the ASD group could show (1) “no fusion”: no integration of either congruent or incongruent cues, like typically developing 6‐year‐old children; this would be consistent with an attenuated priors account [Pellicano & Burr, 2012]; (2) “mandatory fusion”: integration of both congruent and incongruent cues, like typical adults; (3) “selective fusion” in which cues are combined when congruent but not incongruent. This final pattern has not previously been observed but would be predicted by the EPF account of autism.

## Method

### Participants

Twenty three participants with ASD and 15 typically developing adolescents were recruited from two London databases (Birkbeck Babylab and the Centre for Research in Autism and Education) and from an autism unit in a secondary school. All participants had normal or corrected‐to‐normal vision. Inclusion criteria for the typical group included no first‐degree relatives with an autism diagnosis. Participants were excluded for the following reasons: (1) failure to pass the TNO test for stereo vision [Cooper, Feldman, & Medline, 1979]: two ASD participants; (2) failure to complete the task: one ASD participant; (3) any participant with a *d′* score of ≤ 0 (i.e., at or below chance) on any one of the single‐cue conditions *T+*, *D−*, or *D+* (see below): four ASD and one from the typically developing group—this relatively high loss of participants indicates that the difficulty of the task [which matched that previously used with adults; Nardini et al., 2010—see below], was high for these adolescent participants. These exclusions left data for 16 adolescents with ASD (15 male, mean age = 13.8 years) and 14 typically developing adolescents (11 male, mean age = 14.0 years).

All adolescents with autism had received an independent clinical diagnosis of ASD according to DSM‐IV/ICD‐10 criteria. In addition, adolescents scored above the threshold for ASD on either the Autism Diagnostic Observation Schedule—Generic [11 participants completed module 3 and five module 4; Lord et al., 2000] or the Social Communication Questionnaire‐Lifetime [SCQ‐L; Rutter, Bailey, & Lord, 2003], see Table 1.

**Table 1:** Descriptive Statistics

	ASD *M* (SD)	Typically developing *M* (SD)
Age	13.8 (1.2) Range: 12.3–15.9	14.0 (1.3) Range: 12.2–16.3
WASI		
Verbal IQ	99.8 (13.3) Range: 73–119	116.1 (8.5) Range: 101–130
Performance IQ	108.8 (11.9) Range: 88–129	113.5 (8.8) Range: 99–129
Full IQ	104.5 (10.7) Range: 81–124	116.7 (8.3) Range: 103–133
ADOS‐G		
Social‐communication	11.2 (3.5) Range: 4–19	–
Restricted/repetitive	1.2 (1.3) Range: 0–4	–
SCQ total score	25.4 (4.4) Range: 18–35	–

Participants in both groups were administered the Wechsler Abbreviated Scale of Intelligence [WASI; Wechsler, 1999] to index intellectual ability. There was no significant group difference for performance IQ scores, *t*(28) = 1.21, *P* = 0.24, although the groups were not matched on full scale IQ, *t*(28) = 3.44, *P* = 0.002 or verbal IQ, *t*(28) = 3.95, *P* < 0.001. The difference in verbal and full scale IQ scores was due to above average scores in the typical group, rather than below average score in the ASD group (see Table 1). Our ASD group are thus high‐functioning and the results of this study cannot be generalized to lower‐functioning individuals.

### Measures and Procedure

The procedure is identical to that reported for the adult participants in Nardini et al. [2010, Experiment 2]. After the initial TNO test to screen for stereo vision deficits, participants took part in the main study. We wished to establish that all participants had stereo vision, required for completing the stereo‐only conditions, as unlike visual acuity which is commonly corrected, stereo‐vision is not routinely tested or correctable. Participants viewed pairs of elliptical discs presented adjacently on a CRT computer screen at a distance of 175cm (see Fig. 1b–d), with a width of 13° of visual angle. A chin rest lined up each participant's viewing position with the horizontal and vertical center of the screen. LCD shutter glasses (CrystalEyes 3; StereoGraphics) were used to present separate images to the two eyes (each refreshed at 60 Hz). The task was explained and participants were first given 5 practice trials on a randomly selected condition to check they understood the instructions. On each trial a standard disk slanted at 45º, randomly positioned on the left or right, was presented alongside a comparison disc. The comparisons had either the same slant (on half of trials) or a slant differing by ±12.5 degrees (on half of trials). Participants judged whether the discs were the same or different in their slant. The level of slant had been piloted by Nardini et al. [2010] to avoid floor and ceiling effects in typical adult participants.

Discs were comprised either of colored tiles, viewed monocularly (providing only texture information, but not disparity), of dots that had stereo disparities but uniform density on the screen (providing only disparity information, but not texture) or of colored tiles, viewed binocularly (providing both texture and disparity information). The projections to the eyes were those for real objects 16 cm wide, each with depth either +10cm or −10cm relative to the screen, and each with a randomly chosen length between 1 and 1.5 times this width. Tiles were constructed by a Voronoi tessellation around a grid of 1cm‐spaced points (each jittered randomly by ±0–0.225cm); dots by ±0–1.5cm jittering of a grid of 1cm‐spaced points. To allow for a dissociation between disparity‐ and texture‐indicated slant, 3D positions of points were reprojected and calculated to simulate 3D stimuli that have both the texture gradient and the disparities required. All projections were calculated by taking into account each individual participant's interocular distance. Full methods are described in Nardini et al. [2010].

There were 6 conditions, single‐cue conditions *D+* and *D−* (Fig. 1d), *T+* and *T*− (Fig. 1c) and combined cue conditions *T+D+* (congruent) and *T+D*− (incongruent; see Fig. 1b) each with 30 trials, yielding 180 trials in total. In single‐cue conditions *D+*, *D*−*, T+* and *T*−, the slants of both planes were signaled either by disparity (*D*) only or by texture (*T*) only. Condition *T*− was needed to complete this design, but given that this same condition in our earlier study [Nardini et al., 2010] yielded very low scores [as the texture cue to slant becomes increasingly less useful toward the vertical; Hillis, Watt, Landy, & Banks, 2004; Knill, 1998] this condition was not analyzed here (see Supporting Information for scores). The comparison stimulus differed (on half of trials) from the standard in its slant by either −12.5° (“‐” conditions) or +12.5 (“+” conditions).

In combined‐cue conditions *T+D+* and *T+D*−, the slants of both planes were signaled by both disparity and texture. In the “congruent” condition *T+D+*, the comparison stimulus differed (on half of trials) from the standard in its slant by +12.5°. In the “incongruent” condition *T+D*−, the comparison stimulus differed (on half of trials) from the standard in its slant by +12.5° in terms of texture, but by −12.5° in terms of disparity. *D* conditions, *T* conditions and *DT* conditions were presented mixed in pairs (in blocks of 10 trials comprising 5 trials each of a pair, e.g., 5 each of *D+* and *D*−), to avoid biasing participants toward looking for a particular direction of slant difference. A *d*′ sensitivity score was calculated for each condition separately.

### Statistical Analysis and Cue Combination Predictions

Combining (averaging) congruent texture and disparity cues to slant is predicted to show better sensitivity (higher *d*′) in the condition *T+D+* than in either *T+* or *D+*, in which these same cues are presented alone (Fig. 1e). This benefit is predicted by Bayesian cue combination [Clark & Yuille, 1990; Yuille & Bulthoff, 1996] and signal detection theory [Green & Swets, 1966], and has been found both in general [Ernst, 2006] and with these specific depth cues—in adults [Hillis et al., 2002, 2004; Murphy, Ban, & Welchman, 2013, Nardini et al., 2010], but not in typically developing children aged below 12 years [Nardini et al., 2010]. However, combining (averaging) incongruent cues in condition *T+D‐* predicts lower sensitivity (lower *d*′) in this condition than in either *T+* or *D‐*, in which these same cues are presented alone. Intuitively, this is because averaging slant differences of +12.5° and −12.5° via the two cues would lead to some cancelling out and so a percept that could be (if each cue were weighted exactly 50%) of as little as zero slant difference. Therefore, averaging of these conflicting cues would make slant differences on “different” trials appear less than when viewed via either single cue, and so would make the task of distinguishing “different” from “same” slant trials more difficult. This decrement in performance, which has been termed “mandatory fusion” has been seen (with slant stimuli such as these) in adults [Hillis et al., 2002; Nardini et al., 2010], but not in children aged 6 years [Nardini et al., 2010].

When there is a large discrepancy between precision on two consistent single‐cue conditions, the prediction even for an ideal Bayesian observer is that they will obtain minimal benefit by averaging cues as compared with relying on the single more reliable cue. This is because a much less reliable cue is contributing very little useful information to the estimate. To check the difference in performance across unimodal conditions we computed absolute difference scores, that is, *D+* − *T+*, a “congruent difference score” and *D*− − *T+*, an “incongruent difference score.” No significant group differences were found for either the congruent difference score (*T+* − *D+*; ASD mean = 0.55, SD = 1.14; typical mean = −0.06, SD = 1.03; *t*(28) = −1.154, *P* = 0.14) or the incongruent difference score (*T+‐* − *D‐*; ASD mean = 0.58, SD = 0.105; typical mean = −0.33, SD = 1.12; *t*(28) = −0.62, *P* = 0.54). Therefore, the single cue reliabilities and differences in these did not significantly differ across groups in a way that might affect cue combination. However, one participant in the ASD group did have discrepant congruent and incongruent difference scores > 2 SDs above the mean. In the Supporting Information, the main analysis is repeated removing this participant and results remain substantively similar.

Our primary analysis was a set of planned comparisons (paired t‐tests) testing the specific prediction of weighted averaging, that (1) *T+D+* will be higher than both single cues *T+* and *D+* if congruent cues are combined; (2) *T+D*− will be lower than both single cues *T+* and *D*− if incongruent cues are combined (see Fig. 1e). Integration requires both t‐tests to be significant (e.g., in the congruent *T+D+* vs. *T+* and *D+* comparisons, significantly increased performance relative to only one of the single cue conditions would be consistent with the possibility that participants simply rely on their best single cue). Keeping the significance level at the 5% level for each *t*‐test is highly conservative—the probability of Type 1 error on both comparisons (i.e., concluding that *T+D+* is higher than both *T+* and *D+* when is it not is 0.05^2^ = 0.0025).

To examine whether cue combination differed across groups, we calculated a “congruent integration score” and an “incongruent integration score” separately for each participant. The congruent integration score was the difference between the congruent combined cue condition (*T+D+*) and the participant's best single cue (either *T+* or *D+*). Positive scores indicate a precision gain from cue combination (see Fig. 1e). The incongruent integration score was the difference between the incongruent combined cue condition (*T+D*−) and the participant's worst single cue (either *T+* or *D*−). Negative scores indicate a precision loss from cue combination via “mandatory fusion” (see Fig. 1e).

## Results

### Integration of Congruent Cues

**Figure 2:**
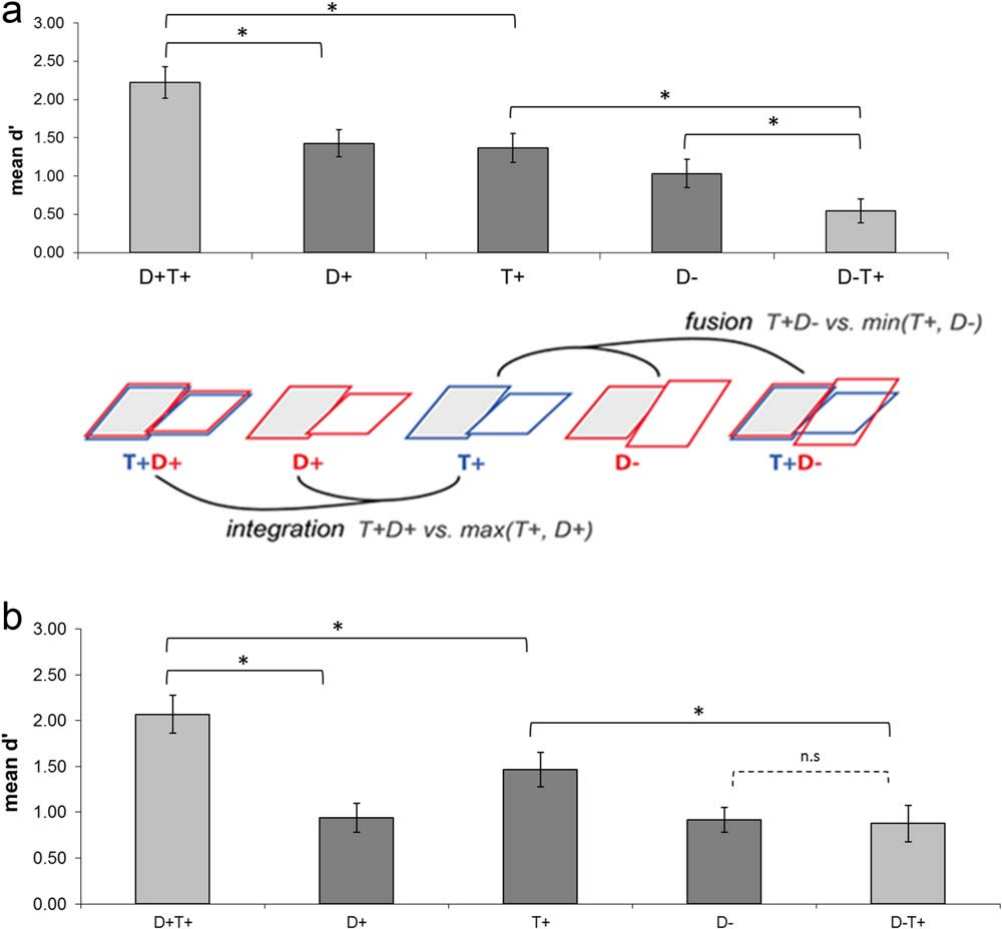
(a) Significant integration of *congruent* cues (*T+D+* vs. *T+* and *D+*) and *incongruent* cues (*T+D*− vs. *T+* and *D−*) in typically developing adolescents and (**b)** Significant integration of *congruent* cues (*T+D+* vs. *T+* and *D+*) but not *incongruent* cues (*T+D*− vs. *T+* and *D*−) in adolescents with ASD.

To assess the integration of congruent cues we compared sensitivity to the combined congruent condition (*T+D+*) with each of the two unimodal conditions (*T+* and *D+*). As expected, typically developing adolescents showed significantly higher *d*′ scores (see Fig. 2a) for the bimodal condition (*T+D+*; mean = 2.23, SE = 0.21) than for the concomitant single‐cue conditions texture (*T+* mean = 1.37, SE = 0.19; *t*(13) = 2.91, *P* = 0.012, Cohen's *d* = 0.78) and disparity (*D+* mean = 1.43, SE = 0.18; *t*(13) = 2.96, *P* = 0.011, Cohen's *d* = 0.79). Consistent with integration, adolescents with autism (Fig. 2b) also showed a significantly greater mean score in the bimodal condition (*T+D+*; mean = 2.18, SE = 0.19) than for both the single cues: texture (*T+*, mean = 1.5, SE = 0.21; *t*(15) = 2.767, *P* = 0.015, Cohen's *d* = 0.39); disparity (*D+*; mean = 0.98, SE = 0.17; *t*(15) = 4.84, *P* < 0.001, Cohen's *d* = 1.21).

### Integration of Incongruent Cues

Integration of incongruent cues predicts lower sensitivity in the combined‐cue condition *T+D*− than in the corresponding unimodal conditions *T+* and *D*−; see Figure 1e. In the typical adolescents, results were consistent with this “mandatory fusion” of incongruent cues, with significantly reduced *d*′ for the bimodal incongruent condition (mean = 0.54, SE = 0.16) compared to *T+* (mean = 1.37, SE = 0.19; *t*(13) = −4.06, *P* = 0.001, Cohen's *d* = 1.09) and *D−* (mean = 1.03, SE = 0.18; *t*(13) = −2.31, *P* = 0.038, Cohen's *d* = 0.62). Adolescents with autism, conversely, did not show this pattern. While performance in the bimodal incongruent condition (*T+D−*) was significantly lower (mean = 0.92, SE = 0.22) than *T+* (mean = 1.54, SE = 0.21), *t*(15) = −2.78, *P* = 0.014, Cohen's *d* = 0.70) no significant difference was found with *D*− (mean = 0.96, SE = 0.5; *t*(15) = −0.18, *P* = 0.86, Cohen's *d* = 0.05; see Fig. 2b).

### Between‐Group Effects

Using the congruent and incongruent integration scores, a 2 × 2 mixed ANOVA was run (condition: congruent vs. incongruent; group: ASD/typical). There was a marginally significant main effect of condition: *F*(1,28) = 4.12, *P* = 0.05, *η*p^2^ = 0.13, with greater discrimination for congruent (mean = 0.41, SD = 0.91 than incongruent (mean = −0.07, SD = 0.74) stimuli. However, there was no main effect of group: *F*(1,28) = 1.16, *P* = 0.29, *η*p^2^ = 0.04 nor was the group*condition interaction significant *F*(1,28) = 0.35, *P* = 0.56, *η*p^2^ = 0.012.

## Discussion

In line with the predictions of EPF, autistic adolescents as well as the typical controls showed significant integration of congruent texture and disparity cues when making slant judgments. Sensitivity was increased for both groups in the combined cue condition compared with either cue presented alone. For the typical group, texture and disparity cues were also integrated when they were not congruent (mandatory fusion), which led to poorer performance for the combined incongruent condition than for the single cues. This is consistent with the behavior of typical adults [Nardini et al., 2010]. The adolescents with autism, however, did not show significantly reduced sensitivity in the incongruent condition; performance was similar to typically developing 6‐year‐olds, as previously reported by Nardini et al. [2010].

The findings suggests that autistic individuals can combine cues when it confers an advantage (e.g., to increase accuracy when congruent) but also keep them separate when combining them would be a disadvantage (e.g., relying on the separate cues when they are incongruent). We term this new pattern of sensory behavior “selective fusion.” However, as the between group ANOVA did not reach significance the group differences in fusion patterns should be interpreted with caution. Intact integration of congruent cues suggests that individuals with autism are able to derive combined global percepts, and do not differ from typically developing adolescents.

Results are consistent with a reduced influence of what EPF theory terms “top‐down feedback,” leading to an increased flexibility in perception [Principle 5; Mottron et al., 2006]. Having less mandatory higher‐order perception is consistent with findings of reduced susceptibility to visual illusions in autism [Brosnan, Scott, Fox, & Pye, 2004; Happé, 1996; although see Ropar & Mitchell, 2001]. One possible implication of such a processing style is atypical category learning; categorisation of new group members involves top‐down processes [Mottron et al., 2006]. Children with autism do not show a “discrimination peak” near category boundaries, and while their categorization accuracy is in line with typical controls, they show slower category learning [Soulières, Mottron, Saumier, & Larochelle, 2007].

While our findings are consistent with reduced mandatory top‐down control, the potential mechanisms underlying such enhanced flexibility are not well specified by EPF. Having altered perceptual priors [Lawson, Rees, & Friston, 2014; Pellicano & Burr, 2012; Van de Cruys, de‐Wit, Evers, Boets, & Wagemans, 2013] could offer a potential mechanistic explanation of the present results. Although a flatter perceptual prior [Pellicano & Burr, 2012] could account for the ability to keep cues separate when incongruent, it does not readily explain the integration of congruent cues. Van de Cruys et al. [2013] in their response to Pellicano and Burr [2012], suggest that rather than being uniformly “flatter,” priors in autism could actually be stronger in some cases. Their “predictive coding” framework [Van de Cruys et al., 2014] suggests that comparison between the brain's prediction and the incoming sensory information generates prediction errors. While the use of such errors is critical for maintaining an accurate representation of incoming stimuli, in a noisy sensory world, such errors can sometimes be uninformative. Van de Cruys et al. [2014] argue that knowing when to ignore prediction errors allows generalizability, but in autism the precision of prediction errors is high and inflexible. This results in a failure to tolerate discrepancies, potentially explaining the ability to keep cues separate when incongruent in our task—that is, there is a mismatch between prior information and sensory input. In the congruent condition, however, there is no discrepancy and so cues are integrated in a typical manner.

Another consideration when interpreting this pattern of results includes the possibility that there might be group differences in the decision making part of the process. In other words, individuals with ASD may have different thresholds at which they make same/different judgments. This is something which could be explored by modeling data from a large number of trials. It is also possible that even in typical development between childhood (no integration of congruent or incongruent cues) and adolescence (integration of congruent and incongruent cues) there is a period where congruent but not incongruent cues are combined. If this is the case, it is possible that the adolescents with ASD are developmentally delayed and are simply showing a typical but delayed pattern. Future studies assessing the integration of congruent and incongruent cues in adults with autism will be required to test this definitively.

One advantage of the stimuli used in the present study is that the basis for mature integration in specific areas of human visual cortex is known [Murphy, Ban, & Welchman, 2013; Welchman, Deubelius, Conrad, Bulthoff, & Kourtzi, 2005]. A recent study using multivariate pattern classification [Murphy et al., 2013] found greater discriminability in area V3B/KO between responses for two different visual slants given combined congruent texture and disparity cues than for either cue alone or incongruent (conflicting) cues. Murphy et al. [2013] demonstrated that when the cues were incongruent activity in V3B/KO was consistent with the behavioral finding of “mandatory fusion” in adults. An important future extension will be test potential neural targets underlying the behavioral differences observed in individuals with ASD.

Relatively small sample sizes are a limitation of this study. While roughly equivalent to previous typical samples tested with this paradigm [Nardini et al., 2010], there is increased variability in our autism group. Although autistic participants often show heterogeneous data, larger studies will be important to detect potential subgroups who may be performing differently. Assessing the generalizability of the present findings is also important. Specifically, are cues combined in this flexible manner (“selective fusion”) when using different pairs of depth cues or other visual cues. And how are conflicting cross‐modal cues integrated? The pattern of sensory integration seen may depend on the modality of the cues, and their neural representation. Assessing integration abilities in low‐functioning autistic individuals will also be necessary to extend the current findings to the broader autistic population. Finally, future work looking at autistic cue integration longitudinally will be interesting to establish the age at which trajectories begin to diverge.

In conclusion, this is the first study to show that adolescents with ASD are able to integrate congruent cues, in line with performance of typical adolescents, but keep separate incongruent cues. Both adults and typical teenagers are subject to mandatory fusion—they cannot help but integrate cues, even when the cues are in conflict. The more flexible ability to combine cues when congruent but keep them separate when incongruent enables adolescents with autism to discriminate stimuli that typical controls cannot. Such perceptual abilities are consistent with the predictions of EPF theory and the more mechanistic predictive coding framework.

## Supporting information

Additional Supporting Information may be found in the online version of this article at the publisher's website:

T‐ condition
Outliers

## Footnotes

1 Specifically, one with heavier tails than a Gaussian—see Ernst and Di Luca [2011] and Ernst [2012] for details.
