# Supplementary material for: Flexible integration of visual cues in adolescents with autism spectrum disorder
Source: Autism Res. 2015 Jun 19;9(2):272–81. doi: 10.1002/aur.1509 (PMC4864758; doi:10.1002/aur.1509)
Supplement: Supplementary file 1 — T‐ condition Outliers [file AUR-9-272-s001.docx]

Supplementary materials

*T- condition*

The additional *T-* condition included to balance the number of trials was not analysed in the main paper because the texture cue becomes less useful as it approached the vertical (Hillis, Watt, Landy & Banks, 2004; Knill, 1998). However, for reference, the adjusted d**´** scores for this condition were 0.15 (SE = 0.13) for the ASD group and 0.32 (SE = 0.23) for the TD group.

*Outliers*

When there is a large discrepancy between precision on two consistent single-cue conditions, the prediction even for an ideal Bayesian observer is that they will obtain minimal benefit by averaging cues as compared with relying on the single more reliable cue. This is because a much less reliable cue is contributing very little useful information to the estimate. To check the difference in performance across unimodal conditions we computed absolute difference scores i.e., *D+* - *T+*, a “congruent difference score” and *D-* - *T+*, an “incongruent difference score”. One individual in the autism group had a difference score >2 SD above the mean for both incongruent and congruent difference scores. This participant unimodal cue adjusted d**´** scores were 3.34 for T+ and only 0.37 for D+ and 0.34 for D-, which may be due to relatively poor stereo vision.

We re-ran the analysis in the autism group after excluding this participant as an outlier, and the results remained substantively similar. Congruent integration: Scores in the congruent cue condition D+T+ remained significant higher than both unimodal cues (D+, p = 0.001; T+, p = 0.006). For the incongruent condition D-T+, while scores were significantly lower than the T+ condition (p = 0.025), there remained no significant difference in comparison to D- (p = 0.44).

|  |  |  |
| --- | --- | --- |
